# Supplementary material for: Development of the Microemulsion Electrokinetic Capillary Chromatography Method for the Analysis of Disperse Dyes Extracted from Polyester Fibers
Source: Molecules. 2022 Oct 17;27(20):6974. doi: 10.3390/molecules27206974 (PMC9609498; doi:10.3390/molecules27206974)
Supplement: Supplementary file 1 [file molecules-27-06974-s001.zip › molecules-1965977-supplementary.pdf]

1. **Supplementary materials**

   1.1. *Preliminary tests of background electrolyte (BGE) components*

**Table S1**. The compositions of tested BGE.

| NAMEKC | |
|---|---|
| **Number** | **BGE composition** |
| **BGE 1** | 10 mM sodium borate and 200 mM SDS in a mixture of DMSO with MeOH (80:20, v/v) |

| MEEKC | | | | | | | |
|---|---|---|---|---|---|---|---|
| | **ME component [weight %]** | | | | | | |
| **Number** | **stock ME** | | | | | **modifiers** | |
| | **oil phase** | **cosurfactant** (butan-1-ol) | **water phase** | **surfactants** | | **organic modifiers** | **cyclodextrin** |
| **ME1** | 0.66% heptane | 6.55% | 87.99% 40 mM borate buffer | 4.87% sodium cholate | | - | - |
| **ME2** | 0.8% n-octane | 6% | 90.2% 40 mM borate buffer | 3% SDS | | - | - |
| **ME3** | 0.8% n-octane | 6% | 90.2% 40 mM borate buffer | 1.5% SDS | 1.5% sodium docusate | - | - |
| **ME4** | 0.8% n-octane | 6% | 90.2% 40 mM borate buffer | 1.5% SDS | 1.5% Brij 35 | - | - |
| **ME5** | 0.8% n-octane | 6% | 80.2% 80 mM borate buffer | 2.25% SDS | 0.75% Tween 20 | 10% isopropanol | - |
| **ME6** | 0.8% n-octane | 6% | 80.2% 25 mM phosphate buffer pH 2.5 | 2.25% SDS | 0.75% Tween 20 | 10% isopropanol | - |
| **ME7** | 0.33% IL | 6% | 95.67% | 2% SDS | 2% Tween 20 | - | - |

| | | | | | | | |
|---|---|---|---|---|---|---|---|
| | | | 40 mM borate buffer | | | | |
| **ME8** | 0.8% n-octane | 6% | 90.2%<br>40 mM borate buffer | 1.5% SDS | 1.5% sodium cholate | - | - |
| **ME8.1** | 0.8% n-octane | 6% | 90.2%<br>40 mM borate buffer | 1.5% SDS | 1.5% sodium cholate | 10% (v/v) isopropanol | - |
| **ME8.2** | 0.8% n-octane | 6% | 90.2% 40 mM borate buffer | 1.5% SDS | 1.5% sodium cholate | 10% (v/v) isopropanol | 25 mM 2-HP-β-CD** |
| **ME8.3** | 0.8% n-octane | 6% | 90.2% 40 mM borate buffer | 1.5% SDS | 1.5% sodium cholate | 10% (v/v) isopropanol | 25 mM 2-HP-γ-CD*** |
| **ME8.4** | 0.8% n-octane | 6% | 90.2% 40 mM borate buffer | 1.5% SDS | 1.5% sodium cholate | 10% (v/v) isopropanol | 12,5 mM 2-HP-β-CD<br>12,5 mM 2-HP-γ-CD |

- no component present,

*IL- ionic liquid, cation: 1-butyl-3-methylimidazolium, anion: bis(trifluoromethylsulfonyl)imid,

**2-HP-β-CD- 2-hydroxypropyl-β-cyclodextrin,

***2-HP-γ-CD- 2-hydroxypropyl-γ-cyclodextrin

**Table S2.** The results obtained in the preliminary studies for applied background electrolytes.

| Number | Resolution | Level of current < 40 μA | Time of analysis < 30 minutes |
|---|---|---|---|
| BGE1 | - | + | - |
| ME1 | - | -- | - |
| ME2 | - | -- | -- |
| ME3 | - | -- | -- |
| ME4 | - | - | + |
| ME5 | + | + | - |
| ME6 | -- | - | + |
| ME7 | - | - | + |
| ME8 | + | - | - |
| ME8.1 | + | + | - |
| ME8.2 | + | + | + |
| ME8.3 | + | + | + |
| ME8.4 | ++ | + | + |

### 1.2. *Optimization of separation conditions by using Doehlert matrix experimental design*

**Table S3**. Values of coefficients of quadric response model developed for optimization of separation conditions.

| Coefficient | Factor | Value ± SE | Significance |
|---|---|---|---|
| $\beta_0$ | | 0.60 ± 0.08 | + |
| $\beta_1$ | $c_{iPr}$ | 0.26 ± 0.03 | + |
| $\beta_2$ | $c_{CD}$ | 0.01 ± 0.03 | - |
| $\beta_3$ | T | -0.01 ± 0.03 | - |
| $\beta_{12}$ | $c_{iPr} \cdot c_{CD}$ | -0.02 ± 0.08 | - |
| $\beta_{13}$ | $c_{iPr} \cdot T$ | 0.38 ± 0.07 | + |
| $\beta_{23}$ | $c_{CD} \cdot T$ | 0.02 ± 0.08 | - |
| $\beta_{11}$ | $c_{iPr}^2$ | -0.35 ± 0.07 | + |
| $\beta_{22}$ | $c_{CD}^2$ | -0.09 ± 0.1 | - |
| $\beta_{33}$ | $T^2$ | 0.05 ± 0.06 | - |

### 1.3. *Analysis of real samples*

Identification of threads from the group (1)- red fabrics

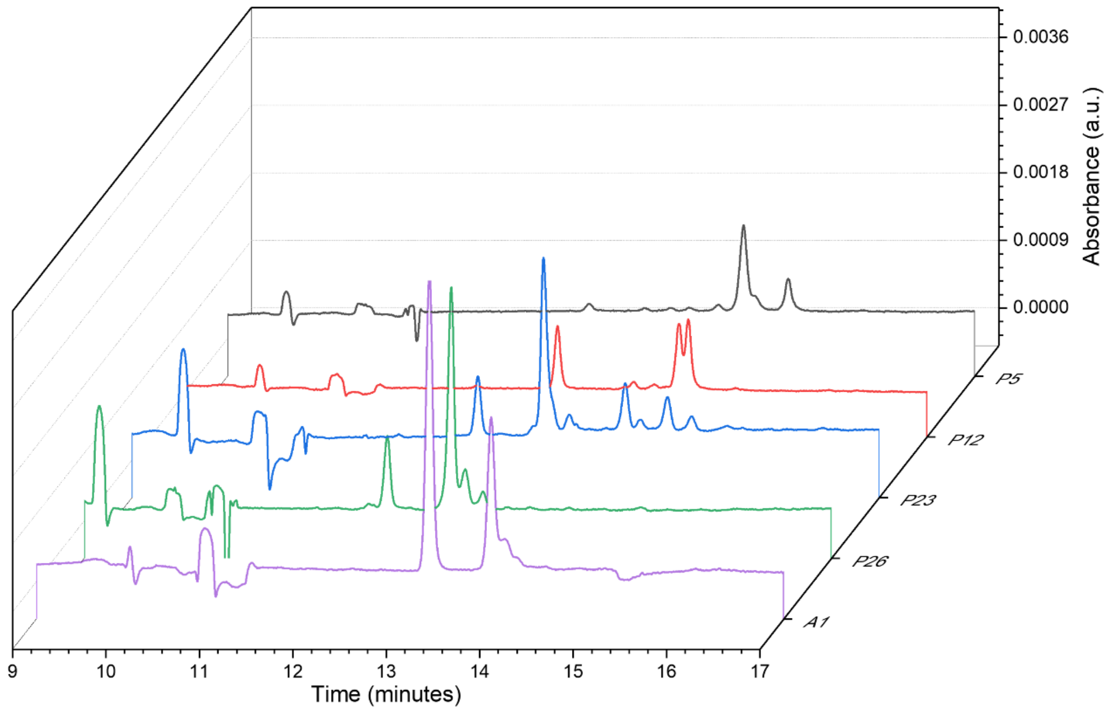


**Figure S1.** Electropherograms with dye signals, recorded at 220 nm for the extracts from fibers included in group (1) from red fabrics and sample A1, secured from one of the textiles.

**Table S4.** Results of differentiation of samples from a group of red fabrics (1) and sample A1; yellow and red colors corresponding to signals with the maximum absorption bands at 380-490 and 490-580, respectively.

| Signal number | relative migration times of the analytes P5 | P12 | P23 | P26 | A1 | *U (k=2)* for samples P26 and A1 |
|---|---|---|---|---|---|---|
| **1** | 1.51 | 1.32 | 1.33 | 1.34 | 1.32 | 0.02 |
| **2** | 1.52 | 1.46 | 1.40 | 1.41 | 1.39 | 0.03 |
| 3 | 1.56 | 1.50 | 1.43 | 1.43 | 1.40 | 0.04 |
| 4 | - | - | 1.50 | - | - | - |
| 5 | | | 1.51 | | | |
| 6 | | | 1.54 | | | |
| 7 | | | 1.57 | | | |
| **total** | 3 | 3 | 7 | 3 | 3 | - |
| **dye** | 3 | 2 | 4 | 2 | 2 | |

The total number of signals excludes the origin common of samples A1 and P23. The number of dye signals excludes similarity with sample P5. The absorption bands in the Vis spectrum of signal number 2 exclude the origin common samples P12 and A1 and indicate the similarity of samples A1 and P26. The calculated values of *U* are lower than 0,11 (the highest value obtained in method validation (see table 6). This led to the conclusion that samples A1 and P26 are secured from the same material.

Identification of threads from the group (3)- blue fabrics.

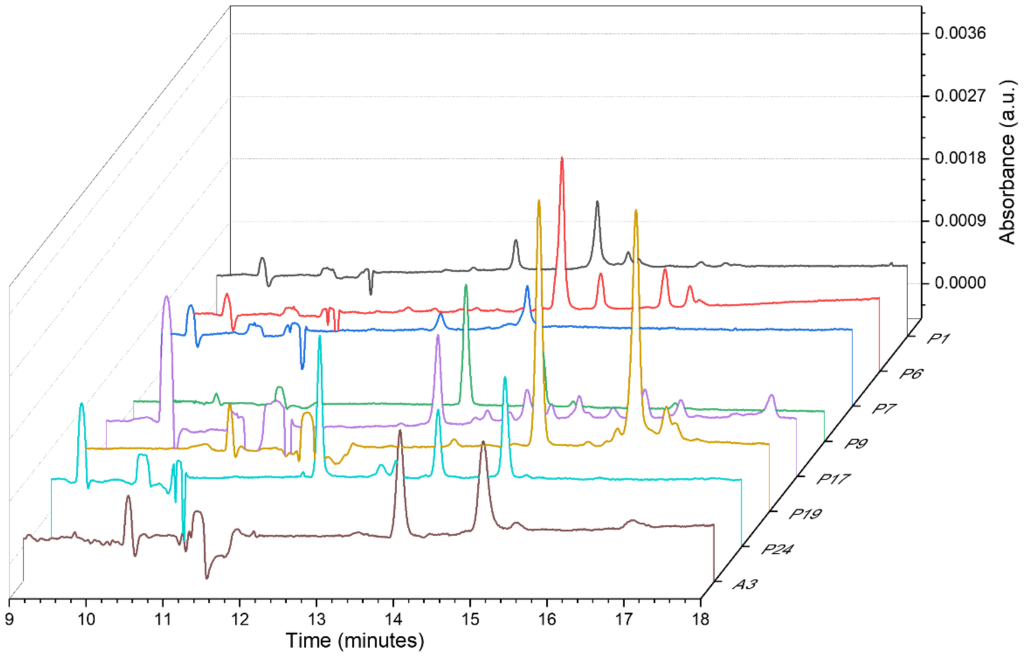


**Figure S2**. Electropherograms with dye signals, recorded at 220 nm for the extracts from fibers included in group (3) from blue fabrics and sample A3, secured from one of the textiles.

**Table S5.** Results of differentiation of samples from a group of blue fabrics (3) and sample A3; yellow, red, and blue colors corresponding to signals with the maximum absorption bands at 380-490, 490-580, and > 580 nm, respectively.

| Signal number | relative migration times of the analytes | | | | | | | | *U* (*k*=2) for samples P9 and A3 |
|---|---|---|---|---|---|---|---|---|---|
| | P1 | P6 | P7 | P9 | P17 | P19 | P24 | A3 | |
| 1 | 1.34 | 1.46 | 1.35 | 1.32 | 1.36 | 1.27 | 1.33 | 1.34 | 0.03 |
| 2 | 1.45 | 1.51 | 1.44 | 1.42 | 1.48 | 1.37 | 1.42 | 1.45 | 0.03 |
| 3 | 1.50 | 1.60 | 1.47 | | 1.51 | 1.42 | 1.44 | | |
| 4 | 1.51 | 1.63 | | | 1.55 | 1.45 | 1.49 | | |
| 5 | | 1.65 | | | 1.64 | 1.46 | 1.59 | | |
| 6 | | | - | - | 1.68 | 1.48 | | - | - |
| 7 | - | - | | | 1.81 | 1.52 | - | | |
| 8 | | | | | - | 1.53 | | | |
| **total** | 4 | 5 | 3 | 2 | 7 | 8 | 5 | 2 | - |
| **dye peaks** | 3 | 3 | 1 | 1 | 0 | 3 | 3 | 1 | |

The total number of signals excludes the origin common of samples A3 with all the samples except P9. The number of dye signals and the absorption bands in the Vis spectrum indicates the similarity of samples A3 and P9. The values of *U* are below 0,11 (the highest value obtained in method validation) (see table 6). This led to the conclusion that samples A3 and P9 are secured from the same material.

Identification of threads from the group (4) - black fabrics.

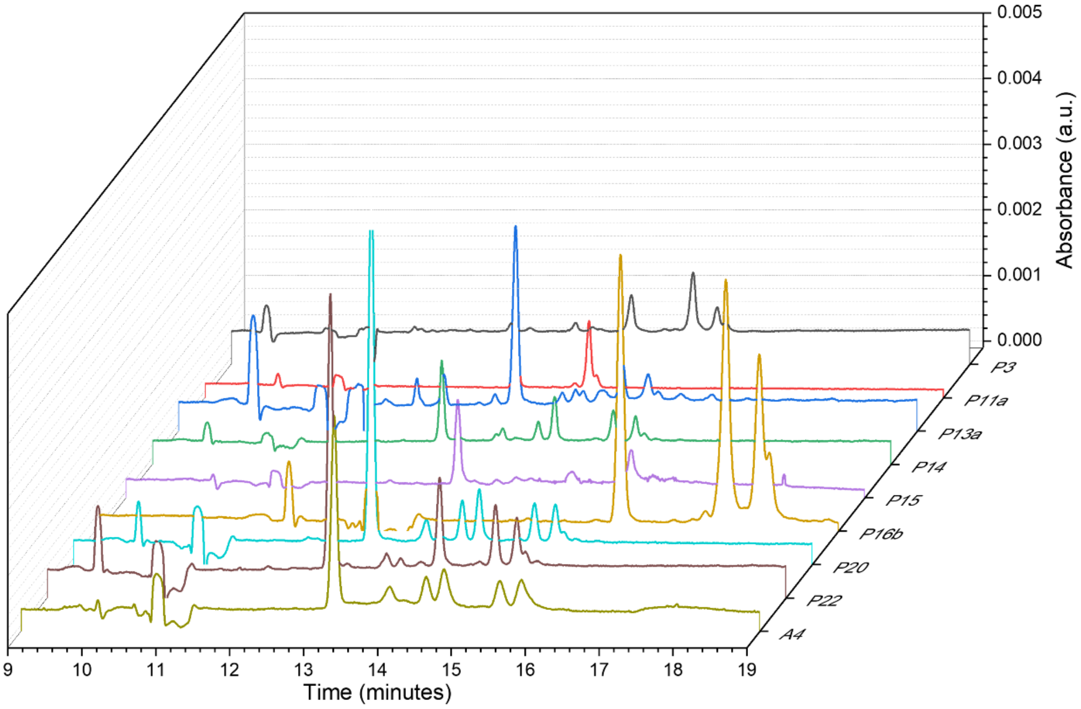


**Figure S3**. Electropherograms with dye signals, recorded at 220 nm for the extracts from fibers included in group (4) from black fabrics and sample A4, secured from one of the textiles.

**Table S6.** Results of differentiation of samples from a group of black fabrics (4) and sample A4; yellow, red, and blue colors corresponding to signals with the maximum absorption bands at 380-490, 490-580, and > 580 nm, respectively.

| signal number | sample symbol | | | | | | | | | *U* (*k*=2) for samples P20 and A4 |
|---|---|---|---|---|---|---|---|---|---|---|
| | P3 | P11a | P13a | P14 | P15 | P16b | P20 | P22 | A4 | |
| 1 | 1.35 | 1.32 | 1.22 | 1.32 | 1.33 | 1.39 | 1.32 | 1.32 | 1.32 | 0.00 |
| 2 | 1.44 | 1.42 | 1.26 | 1.41 | 1.56 | 1.51 | 1.39 | 1.40 | 1.39 | 0.00 |
| 3 | 1.52 | 1.43 | 1.36 | 1.46 | | 1.55 | 1.44 | 1.42 | 1.44 | 0.00 |
| 4 | 1.61 | | 1.44 | 1.48 | | 1.56 | 1.47 | 1.48 | 1.47 | 0.00 |
| 5 | 1.64 | | 1.45 | 1.56 | | | 1.54 | 1.56 | 1.54 | 0.00 |
| 6 | 1.66 | | 1.47 | 1.59 | - | | 1.57 | 1.59 | 1.57 | 0.00 |
| 7 | | - | 1.50 | | | - | | 1.60 | | |
| 8 | - | | 1.53 | - | | | - | | - | - |
| 9 | | | 1.55 | | | | | - | | |
| **total** | 6 | 3 | 9 | 6 | 2 | 4 | 6 | 7 | 6 | - |
| **dye peaks** | 1 | 2 | 1 | 5 | 1 | 3 | 4 | 3 | 4 | |

The total number of signals excludes the similarity of A4 with samples P11a, P13a, P15, P16b, and P22. The number of dye signals excludes the origin common of A4 with samples P3 and P14. The number of dye signals, as so the absorption bands in the Vis spectrum indicate the similarity of A4 and P20. The calculated *U* values are lower than 0.11 (minimum *U* value obtained in the method validation process). This indicates the very good similarity of the samples (see table 6) and leads to the conclusion that samples A4 and P20 are secured from the same source.
